# Charge Injection and Energy Transfer of Surface-Engineered InP/ZnSe/ZnS Quantum Dots

**DOI:** 10.3390/nano13071159

**Published:** 2023-03-24

**Authors:** Jumi Park, Taehee Kim, Dongho Kim

**Affiliations:** 1 Department of Chemistry, Yonsei University, 50 Yonsei-ro, Seodaemun-gu, Seoul 03722, Republic of Korea; 2 Division of Energy Materials, Pohang University of Science and Technology (POSTECH), Pohang 37673, Republic of Korea

**Keywords:** quantum dots, indium phosphide, organic ligands, photophysical properties, electrochemical properties

## Abstract

Surface passivation is a critical aspect of preventing surface oxidation and improving the emission properties of nanocrystal quantum dots (QDs). Recent studies have demonstrated the critical role of surface ligands in determining the performance of QD-based light-emitting diodes (QD-LEDs). Herein, the underlying mechanism by which the capping ligands of InP/ZnSe/ZnS QDs influence the brightness and lifetime of the QD-LEDs is investigated. The electrochemical results demonstrate that highly luminescent InP/ZnSe/ZnS QDs exhibit modulated charge injection depending on the length of the surface ligand chains: short alkyl chains on the ligands are favorable for charge transport to the QDs. In addition, the correlation between the spectroscopic and XRD analyses suggests that the length of the ligand chain tunes the ligand–ligand coupling strength, thereby controlling the inter-QD energy transfer dynamics. The present findings shed new light on the crucial role of surface ligands for InP/ZnSe/ZnS QD-LED applications.

## 1. Introduction

Environmentally benign indium phosphide (InP) quantum dots (QDs) have gained significant attention as a promising platform for commercializing quantum dot light-emitting diodes (QD-LEDs) due to their highly efficient, stable, and color-pure photoluminescence (PL) with a near-unity PL quantum yield and narrow-band emission [1,2,3,4,5,6]. However, the retention of these remarkable properties in electroluminescence (EL) remains challenging [7,8,9,10,11]. The PL and EL each involve the recombination of non-equilibrium charge carriers, but they differ in their charge generation processes. The PL occurs when a material is excited by an external light source, thereby generating non-equilibrium charge carriers that can recombine, resulting in the emission of light. However, EL occurs when a voltage is applied to a material, thereby creating a flow of non-equilibrium charge carriers, such as electrons and holes, that release energy in the form of light when they recombine. Consequently, efficient charge injection and transfer within the device are critical to achieving high EL efficiency in optoelectronic devices. 

While organic capping ligands are generally used to enhance the colloidal stability and protect the QD surfaces against oxidation, surface ligands have also been reported to affect the performance of QD devices by modulating the charge carrier mobility, charge injection, and balance in the spatial distribution of electrons and holes [8,12,13,14,15,16,17,18]. For instance, the performance of the QD device is determined by the operando electrochemical reactions of the organic ligands, and the conductivity and quantum efficiency can be improved via chlorination of the QD surface, which engineers exciton confinement [17,18]. In addition, colloidal core–shell QDs have been shown to exhibit significantly improved QD-LED performance as the surface ligand chain length is reduced [19]. Conversely, QDs with ligands with long chains exhibit lower charge transfer rates due to the large surface transport barrier and the weakening of the charge coupling between QDs [20,21,22,23]. At the same time, the fluorescence resonance energy transfer (FRET) between the QDs is a major determinant of the QD-LED performance particularly the device lifetime [19,24,25]. Energy transfer between QDs can lead to nonradiative recombination, which decreases the device efficiency and shortens its lifetime. This effect is referred to as energy-transfer-induced quenching and is particularly problematic when the QDs are closely packed together. Therefore, controlling energy transfer processes in QD-LEDs is crucial to optimizing the devices’ performance and enhancing their lifetimes. 

Hence, the present study examines the synthesis and electrochemical characterization of InP/ZnSe/ZnS QDs with various surface ligand lengths. Furthermore, the inter-QD electronic energy transfer properties are thoroughly probed via the emerging technique of fluorescence lifetime imaging microscopy (FLIM) by obtaining the PL lifetime and intensity with a scan area of 5 × 5 µm^2^. Since the scanned image has an array of 512 by 512 pixels, the dimensions of each pixel are about 10 × 10 nm^2^. The electrochemical results indicate that decreasing the ligand chain length facilitates charge injection due to a smaller energy barrier towards charge transport, thus highlighting the crucial role of the surface ligand in determining the efficiency of charge injection in the nanoparticles and, in turn, their performance in optoelectronic devices. Moreover, the FLIM results reveal that the energy transfer population and efficiency between neighboring QDs are proportional to the surface ligand chain lengths. In addition, the X-ray diffraction (XRD) patterns of InP/ZnSe/ZnS QD solids prove that the alkyl-chain-length-controlled interligand coupling is a strong driving force for FRET and that shorter alkyl ligands result in weaker interligand van der Waals interactions. Therefore, reducing the surface ligand length of the InP-based QDs improves the performance of the QD-LED by promoting effective charge injection and reducing FRET.

## 2. Materials and Methods

**Scheme 1:**
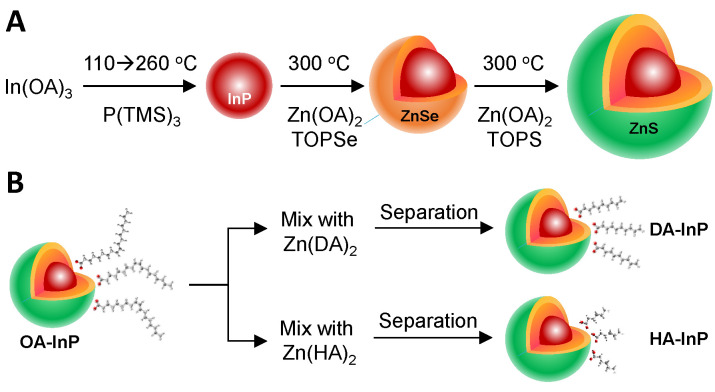
(**A**) The preparation of the InP cores and growth of the ZnSe/ZnS shell. (**B**) The ligand exchange process.

**Figure 1:**
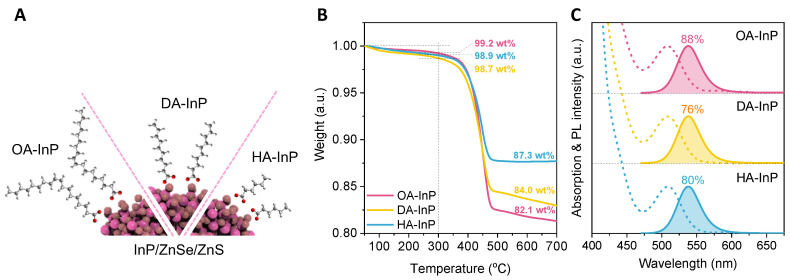
(**A**) A schematic representation of the InP/ZnSe/ZnS QDs with varying lengths of surface-ligands; (**B**) the thermogravimetric analysis and (**C**) the steady-state absorption and photoluminescence spectra of the InP/ZnSe/ZnS QDs before and after the ligand substitution reactions. The values above the spectra indicate the relative quantum yields relative to that of coumarin 153 in ethanol.

As shown in Scheme 1 and Figure 1A, QDs surrounded by oleic acid (OA), decanoic acid (DA), or hexanoic acid (HA) moieties were synthesized using the procedure reported by Hahm et al. with minor modifications [26]. The ligand-exchanged InP/ZnSe/ZnS QDs were prepared via post-synthetic methods to eliminate any experimental errors originating from batch-to-batch variations in the QD synthesis. The as-synthesized InP/ZnSe/ZnS QDs from the same batch were then used to prepare OA-InP, DA-InP, and HA-InP. The full details of starting materials, commercial sources, quantities and synthetic conditions used are provided in the Supplementary Materials.

In addition, the monodispersed QD-ordered OA-InP, DA-InP, and HA-InP films were fabricated by using an established procedure [27,28,29]. The details are provided in the Supplementary Materials. The effects of ligand length upon the energy transfer dynamics of the InP/ZnSe/ZnS QDs were further validated by using a lab-built FLIM setup to investigate the FRET phenomenon. In this setup, a pump beam was focused through a 100× oil-immersion objective onto a small spot of the sample, and an image was obtained based on the difference in the PL decay rate and intensity of the emitting sample. For the FLIM measurements, a 470 nm pulsed laser beam with a repetition frequency of 10 MHz was used as the excitation source, and the highly-ordered QD films were photoexcited under the condition of ⟨N_X_⟩ >> 1.

Thermogravimetric analysis (TGA) revealed that 31% of the OA was replaced by DA in DA-InP, and 54% of the OA was replaced by HA in HA-InP (Figure 1B). Although previous studies have shown that the ligand chain length can impact the overlap of the electron–hole wavefunction [30,31,32], the three InP/ZnSe/ZnS QDs fabricated herein exhibited similar excitonic peak positions, emission energies, and PL quantum yields (Figure 1C). The successful ligand substitution is further demonstrated by the quantitative analysis of the decay curves of the excitonic bleach signal extracted from the transient absorption spectra (Figure S1), in which a fast decay component of a few tens of picoseconds can be attributed to charge transfer into the QD surface. These decay time constants are seen to increase as the ligand chain length of the QDs is extended.

## 3. Results and Discussion

### 3.1. Chronoamperometry

**Figure 2:**
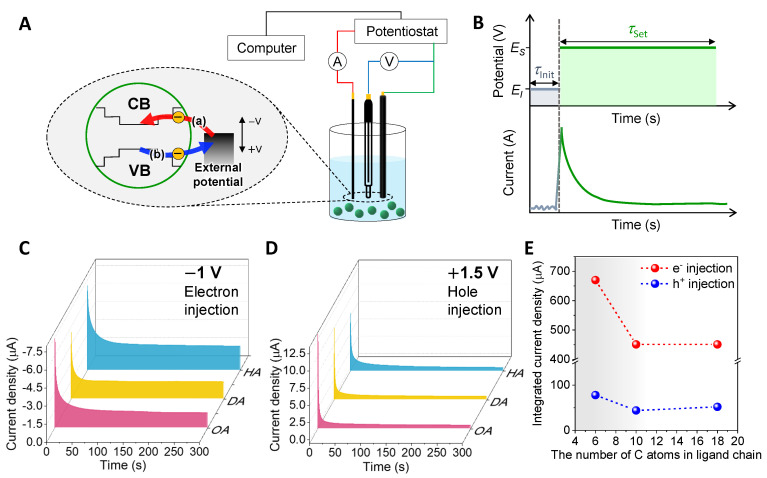
(**A**) A schematic diagram of the electrochemical charging in the QD solution (QD diluted in dichloromethane with 0.05 M TBAPF_6_); (**B**) chronoamperometric measurements, including the potential vs. time profile (top), where *E*_I_ is the initial potential and *E*_S_ is the set applied potential, and the corresponding response (bottom); (**C**,**D**) the chronoamperometric results close to the reduction (**C**) and oxidation (**D**) potential. (**E**) The integrated current density as a function of the number of carbon atoms in the capping ligand.

Considering that charge carriers are electrically generated in the QD-LEDs, the intrinsic charge injection properties of the various InP/Znse/ZnS QDs are examined and compared via chronoamperometry, an electrochemical technique that monitors the current from Faradaic processes occurring at the electrode as a function of time. Because electron migration is poorly modulated, and redox reactions proceed unevenly within QD films [33,34], electrochemical charge injection is performed in the QD solution. A schematic diagram of the charging process is shown in Figure 2A; redox reactions occur in the near-electrode region depending on the external potential. First, the QD energy levels obtained from the onset redox potentials are estimated from the cyclic voltammogram in Figure S3, and the measurement results are summarized in Table S1. The evolution of the current density with time in response to an applied potential is then measured via chronoamperometry, and the results are presented in Figure 2B. Next, the effects of the type of surface ligand on the current density near the onset potential (i.e., −1 and +1.5 V) are revealed in Figure 2C,D. A high current density implies effective charge injection into the corresponding QDs, as it is dependent on the concentration of the charged dots. Thus, the effect of the ligand chain length upon charge injection is seen to be more significant when there are fewer than 10 carbon atoms in the capping ligand (Figure 2E). Decreasing the number of carbon atoms in the ligand from 18 to 6 is seen to increase the integrated current density by a factor of 1.5. In addition, a decrease in ligand length is found to increase the current density at high applied voltages (Figure S4), thereby indicating that short ligands improve the charge injection efficiency by reducing the energy barrier toward charge transport in the InP/ZnSe/ZnS QDs.

### 3.2. Time-Resolved Photoluminescence of Quantum Dot Solids

**Figure 3:**
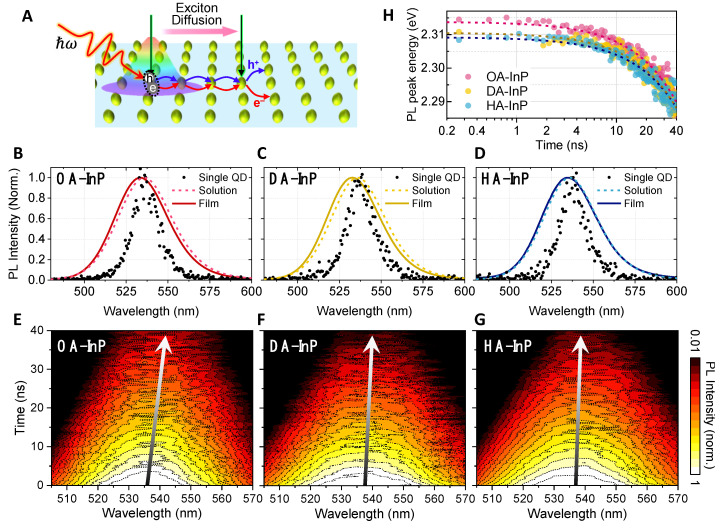
(**A**) A schematic diagram showing the photoinduced energy conversion in QD solids; (**B**–**D**) the photoluminescence (PL) spectra of the OA-InP (**B**), the DA-InP (**C**), and the HA-InP (**D**) in the form of a single QD, a QD solution, and a QD film; (**E**–**G**) the two-dimensional map of time-resolved photoluminescence of the OA-InP (**E**), the DA-InP (**F**), and the HA-InP (**G**) arrays; (**H**) the dynamics of emission peak shifts of the OA-InP, the DA-InP, and the HA-InP arrays.

The performance of the QD-LED is influenced by the interplay between the QDs; hence, it is important to understand the physics of the inter-QD electronic coupling during photoinduced energy conversion, as shown in Figure 3A [35,36,37,38]. Figure 3B−D show the steady-state PL spectra of the OA-InP, DA-InP, and HA-InP, respectively. The single QD emission was recorded with an exposure time of 1 s per frame (see the Supplementary Materials for details on single QD PL microscopy). Zero-dimensional QDs are characterized by the confinement of the electronic wavefunctions in all directions, producing a non-zero electronic density of states only at discrete energies. Thus, narrow PL spectral lines similar to those observed in atomic gases are expected [39]. However, even the spectra of a single QD show significant line broadening due to exciton–phonon coupling, spectral shifts, and exciton fine-structure splitting [40]. The PL spectral linewidth of an ensemble is also affected by inhomogeneous broadening due to particle-to-particle inhomogeneity. In OA-InP, DA-InP, and HA-InP, the slightly increased linewidth of the film compared to the solution suggests an interaction between the QDs. For the QD films, the spectral signature of energy transfer dynamics is revealed by a continuous red-shift of the emission peaks in the two-dimensional (2D) contour maps of the time-resolved PL spectra (Figure 3E−G), accompanied by a decrease in PL intensity [41,42]. Compared to the QD solution and disordered films, a fast-decaying component with a ~2.5 ns time constant appeared in the PL decay kinetics of the QD-ordered films, demonstrating the occurrence of FRET processes (Figures S6 and S7). Additionally, the PL lifetime of each QD array is seen to increase as the emission energy decreases (Figure S8), thereby indicating exciton flow [43]. In particular, the PL lifetime of the OA-InP is seen to increase steeply with the decrease in detection energy, whereas that of the DA-InP and HA-InP varies only modestly with emission energy (Figure S9). Meanwhile, the emission peaks of the OA-InP, DA-InP, and HA-InP arrays exhibit shifts of 30, 24, and 17 meV, respectively, toward low energy (Figure 3H−J). Based on these spectroscopic results, the energy transfer efficiency was expected to vary with ligand length. In general, energy transfer dynamics depend on the donor-to-acceptor separation distance based on the theoretical basis of FRET. However, the transmission electron microscopy (TEM) images reveal similar distances between adjacent QDs of 6.4, 6.3, and 6.2 nm for the OA-InP, DA-InP, and HA-InP arrays, respectively (Figure S5). In addition, the size dispersion is not responsible for the correlation between the FRET kinetics and the ligand chain length given that the QD size distributions were similar regardless of ligand length due to the post-synthetic methods.

### 3.3. Photoluminescence Lifetime Imaging Microscopic Measurements

**Figure 4:**
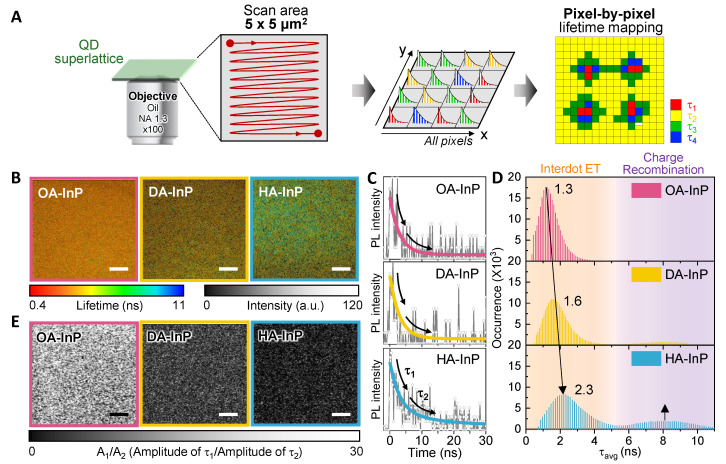
(**A**) A schematic diagram of the fluorescence lifetime imaging microscopy setup; (**B**–**E**) the PL lifetime–intensity images (**B**), the measured (open circles) and double-exponential-fitted (solid lines) cumulative PL decay profiles over the entire scan area (512 by 512 pixels) (**C**), the PL lifetime distributions at each pixel (**D**), and the extracted energy transfer populations from the PL decay curves (**E**) for the OA-InP, DA-InP, and HA-InP. The scale bar corresponds to 1 µm.

To further validate the ligand length dependence of energy transfer dynamics in InP/ZnSe/ZnS QDs, FRET was thoroughly investigated using our lab-built FLIM setup. The FLIM technique provides an image based on the differences in the PL decay rates and intensities from an emitting sample. The 2D digital imaging process is shown schematically in Figure 4A, in which the outcome is an array of pixels defined by the PL decay distribution [44]. Here, each pixel contains lifetime data indicating the number of photons collected at various times after pulsed light excitation. In addition, the pixel-by-pixel PL decay curves provide a sensitive approach to measuring the FRET by quantifying the decrease in the PL lifetime even when the distance between the donor and acceptor QDs is less than 10 nm. The FLIM images of the OA-InP, DA-InP, and HA-InP (scan area: 5.5 × 5.5 µm^2^) are presented in Figure 4B, in which an increase in the PL lifetime is observed with the decrease in ligand length. Meanwhile, the PL decay traces, which represent the total number of photons collected at various times over the entire pixels, exhibit double-exponential decay with a fast-decaying component (τ_1_) reflecting the inter-QD energy transfer processes and a slow-decaying component (τ_2_) reflecting charge recombination (Figure 4C). We further analyzed the FLIM images with an array of 512 by 512 pixels by treating each pixel independently. Figure 4D shows the distribution of PL lifetimes for each pixel. The distribution of the energy transfer rates ($\tau_{\mathrm{FRET}}$) appears within a time window of 0–5 ns (the orange-shaded areas on the left-hand side of Figure 4D). Given that the FRET efficiency (*E*_f_) is proportional to the FRET rate constant ($k_{\mathrm{FRET}}$) [45], the FRET efficiency can be compared using $\tau_{\mathrm{FRET}}$. The average $\tau_{\mathrm{FRET}}$ is seen to increase with the decrease in ligand chain length from 1.3 for the OA-InP to 1.6 and 2.3 ns for the DA-InP and HA-InP, respectively. These experimental results, along with the calculated FRET efficiencies in Table S2, demonstrate that reducing the length of the capping ligand suppresses FRET processes. In addition, the energy transfer populations could be estimated by *A*_1_, where *A*_1_ and *A*_2_ are the amplitude of the fast- (τ_1_) and slow- (τ_2_) decaying components. Figure S10 depicts *A*_1_ and *A*_2_ for 512 by 512 pixels, from which we extracted the numerical results of *A*_1_ over *A*_2_ and visualized them with images in Figure 4E. From these results, the average *A*_1_/*A*_2_ ratios of the OA-InP, DA-InP, and HA-InP are found to be 26.0, 3.33, and 1.03, respectively, thereby indicating that the energy transfer population is significantly decreased as the ligand chain length decreases. Taken together, the spatiotemporal spectroscopic results confirm that the FRET efficiency and population are tuned by the ligand chain length of InP/ZnSe/ZnS QDs.

### 3.4. X-ray Diffraction Spectra and Ligand Ordering

**Figure 5:**
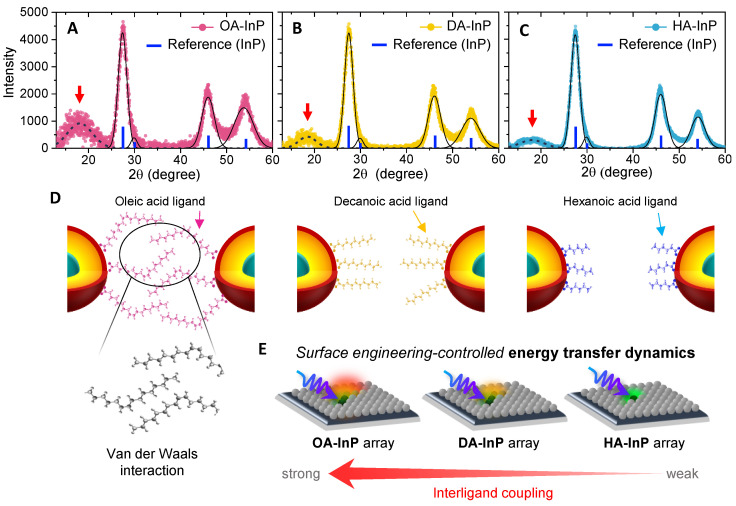
The X-ray diffraction (XRD) patterns of the InP QDs capped with (**A**) OA, (**B**) DA, and (**C**) HA ligands; (**D**) schematic diagrams of the corresponding ligand structures; and (**E**) a schematic illustration of the ligand chain length-controlled inter-QD energy transfer dynamics in InP/ZnSe/ZnS QD-ordered films.

The surface characteristics of the QD samples were investigated using XRD to understand the mechanism by which the ligand length influences inter-QD energy transfer dynamics. The XRD patterns of OA-InP, DA-InP, and HA-InP were experimentally measured and compared with the predicted peaks of zinc-blended InP, represented by blue bars (reference pattern: ISCD 1600543) in Figure 5A−C [46,47,48]. Here, Gaussian peaks were used as an approximation to deconvolute the XRD patterns. In addition to the peaks attributed to InP, an additional unassigned peak was observed around 2θ = 20°, indicated by the red arrow. Alivisatos et al. recently reported that this XRD peak around 2θ = 20° is attributed to aliphatic ligands [46]. 

Interestingly, as the number of carbon atoms in the capping ligand (i.e., the chain length) decrease, the intensity of the ligand peak relative to that of the (111) diffraction peak is seen to decrease, and the ligand peak is seen to shift toward a lower 2θ value (Figure S11). This indicates an increase in the interligand distance—corresponding to weakening van der Waals interactions—as the chain length increases, as shown schematically in Figure 5D. Taken together, these XRD results, along with the qualitative and quantitative interpretations of the spectroscopic features, reveal that the interligand interactions between neighboring QDs significantly affect the FRET efficiency and populations in the InP/ZnSe/ZnS QD films (Figure 5E). The comprehensive results suggest that ligand-length-controlled interligand coupling is an important factor in controlling the FRET process of the colloidal InP/ZnSe/ZnS QDs, which ultimately influences the performance of the QD-LEDs. Notably, previous research on InP/ZnSe/ZnS QD-LEDs has achieved the overall best performance (i.e., excellent EQE and the best lifetime) via ligand displacement from native OA to HA [19].

## 4. Conclusions

The present study investigated the effects of surface ligand upon the optoelectronic properties of InP/ZnSe/ZnS quantum dots (QDs) via electrochemical and time- and space-resolved spectroscopic methods. The results indicated that the surface ligands play crucial roles in both the charge injection and energy transfer processes, which are critical factors for the performance of the QD-based light-emitting diode (LED). Shorter ligands were shown to reduce the barrier towards charge injection, thus leading to an enhanced current flow. Additionally, the spatiotemporal spectroscopic results showed that the rate of inter-QD energy transfer decreased with the decrease in ligand length. From the XRD patterns, we suggested that the interligand van der Waals interactions became weaker with the decrease in the chain length, thus indicating that ligand-length-controlled interligand coupling is a key factor influencing the FRET process in the InP/ZnSe/ZnS QDs. These findings emphasize the importance of surface ligand engineering in optimizing the performance and stability of the QD-LEDs. By tuning the FRET suppression and charge transport promotion in the surface-engineered InP-based QDs, QD-LEDs can achieve high performance and prolonged operational stability.

## Data Availability

The data presented in this study are available on request from the corresponding author.

## Supplementary Materials

The following supporting information can be downloaded at: https://www.mdpi.com/article/10.3390/nano13071159/s1. Figure S1. (A–C) Temporal evolution of the femtosecond transient absorption spectra of OAInP, DA-InP and HA-InP. (D–F) Decay dynamics of bleach signal at 515 nm extracted from (A–C); Figure S2. The photoluminescence intensity monitored at t = 1.5 ns, which is when Auger recombination is completed. Data were fitted with the Poisson statistics. After the lifetime of initial decay of multiexciton, the black solid line indicates fits to (1−p_0_) calculated for the Poisson distribution of initial QD occupancies. These fitted lines were used to determine the absorption cross-section (σ). Obtained absorption cross sections at 2.76 eV are provided as an inset of the Figure (unit: cm^2^); Figure S3. Cyclic voltammograms recorded on OA-InP, DA-InP and HA-InP QDs/dichloromethane containing 0.05 M TBAPF_6_. Scan rates were 200 mVs^−1^; Figure S4. Chronoamperometry measurement at (A) −1.3 V and (B) +1.7 V; Figure S5. Transmission electron microscopy (TEM) images and corresponding histogram of inter QD spacing of (A) OA-InP, (B) DA-InP and (C) HA-InP packing on a TEM grid (Scale bars: 20 nm). (D) Measurements of the inter QD spacing from TEM images using Image J Software; Figure S6. The photoluminescence decay curves of OA-InP, DA-InP and HA-InP solution and solids; Figure S7. (A–C, top) Photoluminescence lifetime-intensity images of disordered InP/ZnSe/ZnS films (Scale bar: 1 μm). (A–C, bottom) Corresponding averaged photoluminescence lifetimes of disordered InP/ZnSe/ZnS films; Figure S8. The photoluminescence decay curves of InP/ZnSe/ZnS QD solids at the wavelengths corresponding to the colored bar in the inset; Figure S9. The photoluminescence lifetimes as a function of detection energy (Inset: fitting parameters representing the rate of increase of the PL lifetime with the detection energy); Figure S10. (A) The images of A_1_ and (B) A_2_ in OA-InP, DA-InP and HA-InP (Scale bar: 1 μm), where photoluminescence decay profiles in FLIM images of OA-InP, DA-InP and HA-InP ordered arrays are fitted using the relation I(t) = A_1_exp(−t/τ_1_) + A_2_exp(−t/τ_2_). I(t) is the time-dependent photoluminescence intensity, A_1_ and A_2_ the amplitudes, and τ_1_ and τ_2_ the fitted photoluminescence lifetimes; Figure S11. (A) Aliphatic ligand peak of XRD spectra of OA-InP, DA-InP, and HA-InP. (B) Relative intensity of ligand peak to the (111) peak and ligand diffraction peak position of InP/ZnSe/ZnS QDs; Figure S12. (A) Deposition of InP/ZnSe/ZnS QDs on the TEM grid by vacuum assisted evaporation of colloidal QD solution (QD dispersed in octane:octanol = 9:1 volume ratio). (B) Growing InP/ZnSe/ZnS QD array on glass substrate by gentle destabilization of the colloidal solution; Table S1. Electrochemical bandgap energies obtained by cyclic voltammogram and optical bandgap energies obtained by absorption spectra; Table S2. FRET efficiency for OA-InP, DA-InP and HA-InP. References [27,35,49,50] are cited in the Supplementary Materials.

## References

[1] Jang E., Kim Y., Won Y.H., Jang H., Choi S.M. (2020). Environmentally Friendly InP-Based Quantum Dots for Efficient Wide Color Gamut Displays. ACS Energy Lett..

[2] Jo J.H., Jo D.Y., Lee S.H., Yoon S.Y., Lim H.B., Lee B.J., Do Y.R., Yang H. (2020). InP-Based Quantum Dots Having an InP Core, Composition-Gradient ZnSeS Inner Shell, and ZnS Outer Shell with Sharp, Bright Emissivity, and Blue Absorptivity for Display Devices. ACS Appl. Nano Mater..

[3] Li Y., Hou X., Dai X., Yao Z., Lv L., Jin Y., Peng X. (2019). Stoichiometry-Controlled InP-Based Quantum Dots: Synthesis, Photoluminescence, and Electroluminescence. J. Am. Chem. Soc..

[4] Jo J.H., Jo D.Y., Choi S.W., Lee S.H., Kim H.M., Yoon S.Y., Kim Y., Han J.N., Yang H. (2021). Highly Bright, Narrow Emissivity of InP Quantum Dots Synthesized by Aminophosphine: Effects of Double Shelling Scheme and Ga Treatment. Adv. Opt. Mater..

[5] Taylor D.A., Teku J.A., Cho S., Chae W.S., Jeong S.J., Lee J.S. (2021). Importance of Surface Functionalization and Purification for Narrow FWHM and Bright Green-Emitting InP Core-Multishell Quantum Dots via a Two-Step Growth Process. Chem. Mater..

[6] Wu Q., Cao F., Wang S., Wang Y., Sun Z., Feng J., Liu Y., Wang L., Cao Q., Li Y. (2022). Quasi-Shell-Growth Strategy Achieves Stable and Efficient Green InP Quantum Dot Light-Emitting Diodes. Adv. Sci..

[7] Li L., Luo Y., Wu Q., Wang L., Jia G., Chen T., Zhang C., Yang X. (2023). Efficient and Bright Green InP Quantum Dot Light-Emitting Diodes Enabled by a Self-Assembled Dipole Interface Monolayer. Nanoscale.

[8] Park S.A., Jung W.H., Yoo J.Y., Lee C.W., Kim J.S., Kim J.G., Chin B.D. (2020). Electrical Resonant Effects of Ligands on the Luminescent Properties of InP/ZnSeS/ZnS Quantum Dots and Devices Configured Therefrom. Org. Electron..

[9] Chao W.C., Chiang T.H., Liu Y.C., Huang Z.X., Liao C.C., Chu C.H., Wang C.H., Tseng H.W., Hung W.Y., Chou P.T. (2021). High Efficiency Green InP Quantum Dot Light-Emitting Diodes by Balancing Electron and Hole Mobility. Commun. Mater..

[10] Shin D.H., Lampande R., Kim S.J., Jung Y.H., Kwon J.H. (2022). Understanding the Origin of Degradation of InP-Quantum Dot Light-Emitting Diodes. Adv. Electron. Mater..

[11] Iwasaki Y., Motomura G., Ogura K., Tsuzuki T. (2020). Efficient Green InP Quantum Dot Light-Emitting Diodes Using Suitable Organic Electron-Transporting Materials. Appl. Phys. Lett..

[12] Jiang Y., Cui M., Li S., Sun C., Huang Y., Wei J., Zhang L., Lv M., Qin C., Liu Y. (2021). Reducing the Impact of Auger Recombination in Quasi-2D Perovskite Light-Emitting Diodes. Nat. Commun..

[13] Heuer-Jungemann A., Feliu N., Bakaimi I., Hamaly M., Alkilany A., Chakraborty I., Masood A., Casula M.F., Kostopoulou A., Oh E. (2019). The Role of Ligands in the Chemical Synthesis and Applications of Inorganic Nanoparticles. Chem. Rev..

[14] Kirkwood N., Monchen J.O.V., Crisp R.W., Grimaldi G., Bergstein H.A.C., Du Fossé I., Van Der Stam W., Infante I., Houtepen A.J. (2018). Finding and Fixing Traps in II-VI and III-V Colloidal Quantum Dots: The Importance of Z-Type Ligand Passivation. J. Am. Chem. Soc..

[15] Wang L., Lv Y., Lin J., Zhao J., Liu X., Zeng R., Wang X., Zou B. (2021). Surface Organic Ligand-Passivated Quantum Dots: Toward High-Performance Light-Emitting Diodes with Long Lifetimes. J. Mater. Chem. C.

[16] Ding S., Hao M., Lin T., Bai Y., Wang L. (2022). Ligand Engineering of Perovskite Quantum Dots for Efficient and Stable Solar Cells. J. Energy Chem..

[17] Shen H., Cao W., Shewmon N.T., Yang C., Li L.S., Xue J. (2015). High-Efficiency, Low Turn-on Voltage Blue-Violet Quantum-Dot-Based Light-Emitting Diodes. Nano Lett..

[18] Li X., Zhao Y.B., Fan F., Levina L., Liu M., Quintero-Bermudez R., Gong X., Quan L.N., Fan J., Yang Z. (2018). Bright Colloidal Quantum Dot Light-Emitting Diodes Enabled by Efficient Chlorination. Nat. Photonics.

[19] Won Y.-H., Cho O., Kim T., Chung D.-Y., Kim T., Chung H., Jang H., Lee J., Kim D., Jang E. (2019). Highly Efficient and Stable InP/ZnSe/ZnS Quantum Dot Light-Emitting Diodes. Nature.

[20] Peng Z., Liu Y., Wu L., Zhao Y., Chen K., Chen W. (2016). Influence of Surface States of CuInS2 Quantum Dots in Quantum Dots Sensitized Photo-Electrodes. Appl. Surf. Sci..

[21] Zeng S., Li Z., Tan W., Si J., Huang Z., Hou X. (2022). Ultrafast Electron Transfer Dynamics Affected by Ligand Chain Length in InP/ZnS Core/Shell Quantum Dots. J. Phys. Chem. C.

[22] Yu S., Fan X.B., Wang X., Li J., Zhang Q., Xia A., Wei S., Wu L.Z., Zhou Y., Patzke G.R. (2018). Efficient Photocatalytic Hydrogen Evolution with Ligand Engineered All-Inorganic InP and InP/ZnS Colloidal Quantum Dots. Nat. Commun..

[23] Smith J.G., Jain P.K. (2016). The Ligand Shell as an Energy Barrier in Surface Reactions on Transition Metal Nanoparticles. J. Am. Chem. Soc..

[24] Pietryga J.M., Park Y.S., Lim J., Fidler A.F., Bae W.K., Brovelli S., Klimov V.I. (2016). Spectroscopic and Device Aspects of Nanocrystal Quantum Dots. Chem. Rev..

[25] Yang Z., Gao M., Wu W., Yang X., Sun X.W., Zhang J., Wang H.C., Liu R.S., Han C.Y., Yang H. (2019). Recent Advances in Quantum Dot-Based Light-Emitting Devices: Challenges and Possible Solutions. Mater. Today.

[26] Hahm D., Chang J.H., Jeong B.G., Park P., Kim J., Lee S., Choi J., Kim W.D., Rhee S., Lim J. (2019). Design Principle for Bright, Robust, and Color-Pure InP/ZnSe*_x_*S_1−*x*_/ZnS Heterostructures. Chem. Mater..

[27] Yoon S.J., Guo Z., Dos Santos Claro P.C., Shevchenko E.V., Huang L. (2016). Direct Imaging of Long-Range Exciton Transport in Quantum Dot Superlattices by Ultrafast Microscopy. ACS Nano.

[28] Talapin D.V., Lee J.S., Kovalenko M.V., Shevchenko E.V. (2010). Prospects of Colloidal Nanocrystals for Electronic and Optoelectronic Applications. Chem. Rev..

[29] Talapin D.V., Shevchenko E.V., Murray C.B., Titov A.V., Kral P. (2007). Dipole-Dipole Interactions in Nanoparticle Superlattices. Nano Lett..

[30] Kroupa D.M., Vörös M., Brawand N.P., McNichols B.W., Miller E.M., Gu J., Nozik A.J., Sellinger A., Galli G., Beard M.C. (2017). Tuning Colloidal Quantum Dot Band Edge Positions through Solution-Phase Surface Chemistry Modification. Nat. Commun..

[31] Boles M.A., Ling D., Hyeon T., Talapin D.V. (2016). The Surface Science of Nanocrystals. Nat. Mater..

[32] Jiang F., Li Y., Ye M., Fan L., Ding Y., Li Y. (2010). Ligand-Tuned Shape Control, Oriented Assembly, and Electrochemical Characterization of Colloidal Znte Nanocrystals. Chem. Mater..

[33] Ashokan A., Mulvaney P. (2021). Spectroelectrochemistry of Colloidal CdSe Quantum Dots. Chem. Mater..

[34] Liu Y., Gibbs M., Ihly R., Law M., Tolentino J. (2011). The Photothermal Stability of PbS Quantum Dot Solids. ACS Nano.

[35] Kagan C.R., Murray C.B., Bawendi M.G. (1996). Long-Range Resonance Transfer of Electronic Excitations in Close-Packed CdSe Quantum-Dot Solids. Phys. Rev. B.

[36] Kholmicheva N., Moroz P., Bastola E., Razgoniaeva N., Bocanegra J., Shaughnessy M., Porach Z., Khon D., Zamkov M. (2015). Mapping the Exciton Diffusion in Semiconductor Nanocrystal Solids. ACS Nano.

[37] Kholmicheva N., Moroz P., Eckard H., Jensen G., Zamkov M. (2017). Energy Transfer in Quantum Dot Solids. ACS Energy Lett..

[38] Moroz P., Romero L.R., Zamkov M. (2019). Colloidal Semiconductor Nanocrystals in Energy Transfer Reactions. Chem. Commun..

[39] Gellen T.A., Lem J., Turner D.B. (2017). Probing Homogeneous Line Broadening in CdSe Nanocrystals Using Multidimensional Electronic Spectroscopy. Nano Lett..

[40] Cui J., Beyler A.P., Coropceanu I., Cleary L., Avila T.R., Chen Y., Cordero J.M., Heathcote S.L., Harris D.K., Chen O. (2016). Evolution of the Single-Nanocrystal Photoluminescence Linewidth with Size and Shell: Implications for Exciton-Phonon Coupling and the Optimization of Spectral Linewidths. Nano Lett..

[41] Mićić O.I., Jones K.M., Cahill A., Nozik A.J. (1998). Optical, Electronic, and Structural Properties of Uncoupled and Close-Packed Arrays of InP Quantum Dots. J. Phys. Chem. B.

[42] Crooker S.A., Hollingsworth J.A., Tretiak S., Klimov V.I. (2002). Spectrally Resolved Dynamics of Energy Transfer in Quantum-Dot Assemblies: Towards Engineered Energy Flows in Artificial Materials. Phys. Rev. Lett..

[43] Baranov D., Fieramosca A., Yang R.X., Polimeno L., Lerario G., Toso S., Giansante C., De Giorgi M., Tan L.Z., Sanvitto D. (2021). Aging of Self-Assembled Lead Halide Perovskite Nanocrystal Superlattices: Effects on Photoluminescence and Energy Transfer. ACS Nano.

[44] Roberts M.S., Dancik Y., Prow T.W., Thorling C.A., Lin L.L., Grice J.E., Robertson T.A., König K., Becker W. (2011). Non-Invasive Imaging of Skin Physiology and Percutaneous Penetration Using Fluorescence Spectral and Lifetime Imaging with Multiphoton and Confocal Microscopy. Eur. J. Pharm. Biopharm..

[45] Zheng K., Žídek K., Abdellah M., Zhu N., Chábera P., Lenngren N., Chi Q., Pullerits T. (2014). Directed Energy Transfer in Films of CdSe Quantum Dots: Beyond the Point Dipole Approximation. J. Am. Chem. Soc..

[46] Calvin J.J., Sedlak A.B., Crook M.F., Alivisatos A.P. (2021). Observation of Ordered Organic Capping Ligands on Semiconducting Quantum Dots via Powder X-Ray Diffraction. Nat. Commun..

[47] Kapadia R., Yu Z.H., Wang H.H., Zheng M., Battaglia C., Hettick M., Kiriya D., Takei K., Lobaccaro P., Beeman J.W. (2013). A Direct Thin-Film Path towards Low-Cost Large-Area III-V Photovoltaics. Sci. Rep..

[48] Patzke G.R., Kontic R., Shiolashvili Z., Makhatadze N., Jishiashvili D. (2013). Hydrazine-Assisted Formation of Indium Phosphide (InP)-Based Nanowires and Core-Shell Composites. Materials.

[49] Makarov N.S., Guo S., Isaienko O., Liu W., Robel I., Klimov V.I. (2016). Spectral and Dynamical Properties of Single Excitons, Biexcitons, and Trions in Cesium-Lead-Halide Perovskite Quantum Dots. Nano Lett..

[50] Lakovicz J.R. (2006). Principles of fluorescence spectroscopy.

