# Supplementary material for: Charge Injection and Energy Transfer of Surface-Engineered InP/ZnSe/ZnS Quantum Dots
Source: Nanomaterials (Basel). 2023 Mar 24;13(7):1159. doi: 10.3390/nano13071159 (PMC10096696; doi:10.3390/nano13071159)
Supplement: Supplementary file 1 [file nanomaterials-13-01159-s001.zip › nanomaterials-2266154-supplementary.pdf]

nanomaterials

MDPI

Supplementary Materials

Charge Injection and Energy Transfer of Surface-Engineered InP/ZnSe/ZnS Quantum Dots

Jumi Park [1,2], Taehee Kim [1] and Dongho Kim [1,2,*]

[1] Department of Chemistry, Yonsei University, 50 Yonsei-ro, Seodaemun-gu, Seoul 03722, Republic of Korea.
[2] Division of Energy Materials, Pohang University of Science and Technology (POSTECH), Pohang 37673, Republic of Korea.
* Correspondence: dongho@yonsei.ac.kr; Tel.: +82-2-2123-2652

Table of Contents

Experimental

1. Steady-State Absorption and Photoluminescence Measurements
2. Cyclic Voltammetry Measurements
3. Picosecond Time-Resolved Photoluminescence Decay
4. Fluorescence Lifetime Imaging Microscopy
5. Single Dot Measurements
6. InP Core Synthesis
7. Preparation of InP/ZnSe/ZnS QDs
8. Ligand Displacement
9. Preparation of QD Array

Supporting Figures

1. Femtosecond transient absorption spectra
2. Absorption cross-section of QDs
3. Cyclic voltammograms
4. Electrical and optical bandgap energies
5. Current densities upon electrochemical charging
6. Transmission electron microscopy (TEM) images of ordered QD films
7. The photoluminescence decay curves of QD solution and array
8. Fluorescence lifetime imaging microscopy (FLIM) images of disordered QD films

9. Fluorescence resonance energy transfer (FRET) efficiency

10. QD array fabrication

Experimental

1. *Steady-State Absorption and Photoluminescence Measurements*

Steady-state absorption spectra were obtained by a UV/Vis spectrometer (Cary 5000, Varian, Palo Alto, CA, USA).

Photoluminescence spectra of QDs were taken with a fluorescence spectrophotometer (F-7000, Hitachi, Chiyoda, Japan). Photoluminescence spectra are spectrally corrected by using correction factor of the fluorescence spectrophotometer. All steady-state measurements carried out by using a quartz cuvette with a pathlength of 1 cm.

2. *Cyclic voltammetry measurements*

The electrochemical setup consisted of a CHI621D potentiostat (CH Instruments, Inc., Bee Cave, TX, USA) with a Pt working electrode, a Pt wire counter electrode and a 0.1 m Ag/AgCl reference electrode. The Ag/AgCl reference electrode (−4.80 V versus vacuum) was calibrated with a ferrocene/ferrocenium couple. In an Ar purged glovebox, the QD solution with an electrolyte consisting of anhydrous dichloromethane (≥99.8%, Sigma-Aldrich, Munich, Germany) and either 0.05 M $TBAPF_6$ (Tetrabutylammonium hexafluorophosphate, for electrochemical analysis, ≥99.0%, Sigma Aldrich) was put in a C-3 low volume cell (Bioanalytical Systems, Inc., West Lafayette, IN, USA). Cyclic voltammograms were measured at a 0.2 Vs^{-1} scan rate and 10^{-4} sensitivity. All chemicals were used as received.

3. *Picosecond Time-Resolved Photoluminescence Decay*

A photoluminescence decay was measured using time-correlated single-photon-counting (TCSPC) system. A mode-locked Ti:sapphire laser (MaiTai BB, Spectra Physics, Andover, MA, USA) was used as an excitation light source, which provides ultrashort pulse with high repetition rate (80 MHz). The high repetition rate was reduced to 800 kHz by using homemade pulse picker. The pulse picked output was doubled in frequency by a 1-mm-thick BBO crystal (type-I, θ = 29.2°, EKSMA). The fluorescence was collected by a microchannel plate photomultiplier (R3809U-51, Hamamatsu, Shizuoka-ken, Japan) connected to a TCSPC board (SPC-130, Becker & Hickl GmbH, Berlin, Germany).

The overall instrumental response function was about 25 ps.

4. *Fluorescence Lifetime Imaging Microscopy*

A spatial distribution of time-resolved photoluminescence decay was recorded using timecorrelated single-photon-counting (TCSPC) system combined with Galvano-scanning system. A mode-locked Ti:sapphire laser (Chameleon Ultra, Coherent, Santa Clara, CA, USA) was used as an excitation light source, which provides ultrashort pulse (140 fs full width at half-maximum at 800 nm) with high repetition rate (80 MHz). The high repetition rate was reduced to 10 MHz by using a pulse picker (pulseSelect, APE, Berlin, Germany). The pulse-picked output was doubled in frequency by a harmonic generator (Harmonics SHG, APE, Berlin, Germany) to generate 470 nm excitation from 940 nm laser. The laser beam was positioned on Galvano scanner (GVS012, Thorlabs, Newton, NJ, USA) with two motorized mirrors connected to Galvano controller (GVD-120, Thorlabs, Newton, NJ, USA). The samples were excited with a 470 nm. The emission was collected through oil immersion objective (UPLFLN, 1.3 NA, 100×, Olympus, Shinjuku, Japan). The photoluminescence signal was collected by the same objective, passed through a dichroic mirror (T470lpxr, Chroma Technology, Bellows Falls, VT, USA), spectrally filtered using a notch filter (HNPF-470.0-1.0, Kaiser optical systems, Ann Arbor, MI, USA) and a long

bandpass filter (FF01-496/LP-25, Semrock, West Henrietta, NY, USA). The photoluminescence was detected by an avalanche photodiode (APD) module (SPCM-AQR-14-FC, EG&G, Boston, MA, USA). The photoluminescence signal detected by the APD was registered by TCSPC PC card (SPC-160, Becker & Hickl, Berlin, Germany). The photoluminescence lifetime image was scanned and constructed in first-in-first-out imaging mode. The overall instrumental response function was about 250 ps in full width at half-maximum. Acquisition time was 20 min. The collected signals were processed by SPC Image software (SPCImage NG Data Analysis Software, version 8.7, Becker & Hickl, Berlin, Germany).

5. *Single Dot Measurements*

Samples for single dot confocal measurements were prepared by spin-coating of QD solutions on coverslips in a nitrogen-filled glove box. The QD solutions were composed of QDs in toluene containing 20 mg/mL polystyrene. A confocal microscope based on an TE2000-U, Nikon was equipped with a sample scanning stage. Circular polarized light from a picosecond pulsed diode laser (LDH-P-C-450B, Picoquent (Berlin, Germany), 1 MHz repetition rate, prepared using a Berek compensator (5540, New Focus, San Jose, CA, USA)) was used to excite the QD samples. The emission was collected through oil immersion objective (Plan Fluor, 1.3 NA, 100×, Nikon, Minato, Japan). The photoluminescence signals were passed through a dichroic mirror (Z457rdc, Chroma Technology, Bellows Falls, VT, USA), spectrally filtered using a notch filter (HNPF-450.0-1.0, Kaiser optical systems, Ann Arbor, MI, USA) and a long bandpass filter (LP02-473RU-25, Semrock, West Henrietta, NY, USA). The fluorescence was detected by an avalanche photodiode (APD) module (SPCM-AQR-16-FC, EG&G, Boston, MA, USA). It was dispersed via a spectrograph (SpectraPro 2150i, Princeton Instruments, Thousand Oaks, CA, USA) and imaged by an EMCCD camera (PL PROEM:512B, Princeton Instruments, Thousand Oaks, CA, USA). The fluorescent signal detected by the APD was registered by a time-correlated single photon counting (SPC 830, Becker & Hickl, Berlin, Germany). The TCSPC was operated in first-in first-out regime in which the arrival time after the beginning of acquisition and the time lag with respect to the excitation pulse were stored for each detected photon. The full-width half maximum (FWHM) of the overall instrumental response function approximately corresponded to 500–600 ps. The data were processed by using a BIFL data analyzer software (BIFLDA, version 1.2, Scientific Software Technologies Center, Minsk, Belarus) to obtain fluorescence intensity trajectories and the time-resolved fluorescence decays.

6. *InP Core Synthesis*

Indium phosphide nanocrystals were synthesized under a nitrogen-filled glovebox atmosphere using air-free Schlenk line techniques. $In(OA)_3$ were prepared by degassing indium acetate (30 mmol) and oleic acid (90 mmol) diluted in 450 ml of ODE under vacuum at 110 °C for 6 hours. $P(TMS)_3$ (15 mmol) dissolved in TOP (30 ml) was injected into the reaction mixture, which was then heated up to 260 °C in 15 min. Synthesized InP QDs were purified twice via precipitation (acetone)/redispersion (toluene) method. The resulting InP core was diluted in toluene.

7. *Preparation of InP/ZnSe/ZnS Quantum Dots*

$Zn(OA)_2$ (7 mmol) dissolved in ODE (20 ml) was degassed at 110 °C and heated to 200 °C. InP core dissolved in toluene (0.5 ml) was injected into the reaction mixture. Then a mixture of TOPSe (0.8 ml of 2 M stock solution) was added stepwise into the reaction flask. To grow ZnS shell, a equilvalent amount of $Zn(OA)_2$ (2.8 mmol) and TOPS (2.8 mmol) was injected to above reaction solution. Synthesized QDs were purified repeatedly via precipitation (acetone)/redispersion(toluene) method, dissolved in toluene. The reaction time for the growth of each shell layer was 2 hours.

8. *Ligand Displacement*

As-synthesized InP/ZnSe/ZnS QDs are stabilized with native oleic acid ligands (OA-InP). For ligand exchange oleic acid for decanoic acid, $Zn(DA)_2$ was prepared following the same procedure as that used to prepare the $Zn(OA)_2$ precursor. Finally, 2.2 mmol of $Zn(HA)_2$ was mixed with 1 g of the OA-InP powder, which was stirred at room temperature for 2 h to yield clear QD dispersion. Resulting DA-InP QDs were purified via the precipitation/redispersion method. HA-InP was obtained using the same separation method as above.

9. *Preparation of QD Ordered Films*

We fabricated ordered-QD arrays following the precedure reported in ref. [27]. As-prepared InP/ZnSe/ZnS QDs were dispersed in toluene and preserved in an N_2-filled glove box. To image the packing behavior of InP/ZnSe/ZnS QDs on TEM grid, as-prepared InP/ZnSe/ZnS QDs are dispersed in octane/octanol (9:1 volume ratio) solution and evaporate solvent in vacuum oven at 80°C as shown in Figure S10(A). For growth the InP/ZnSe/ZnS QD array on glass substrate, we used gentle destabilization of the colloidal solution as shown in Figure S12(B).

Supporting Figures

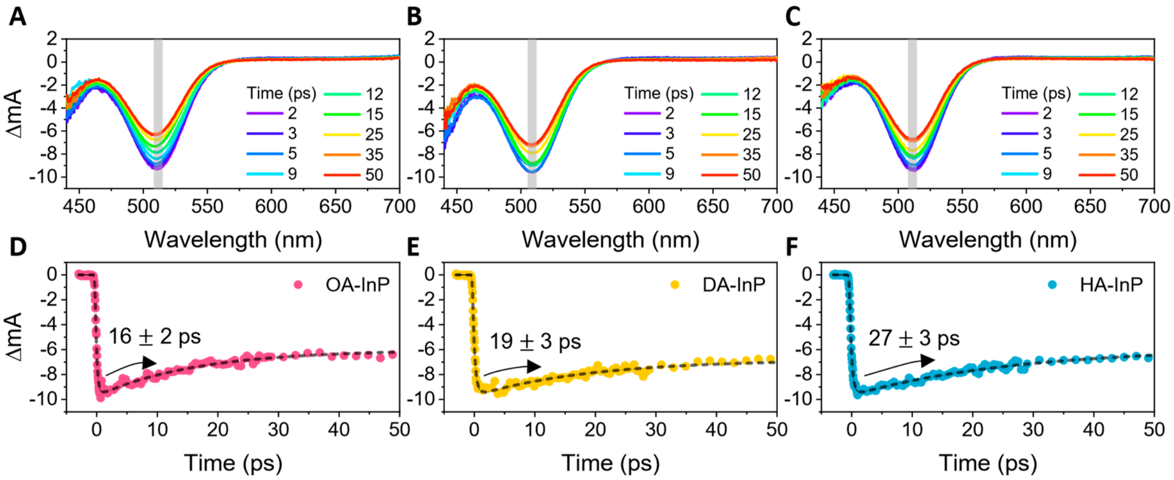

Figure S1. (**A**–**C**) Temporal evolution of the femtosecond transient absorption spectra of OAInP, DA-InP and HA-InP. (**D**–**F**) Decay dynamics of bleach signal at 515 nm extracted from (**A**–**C**).

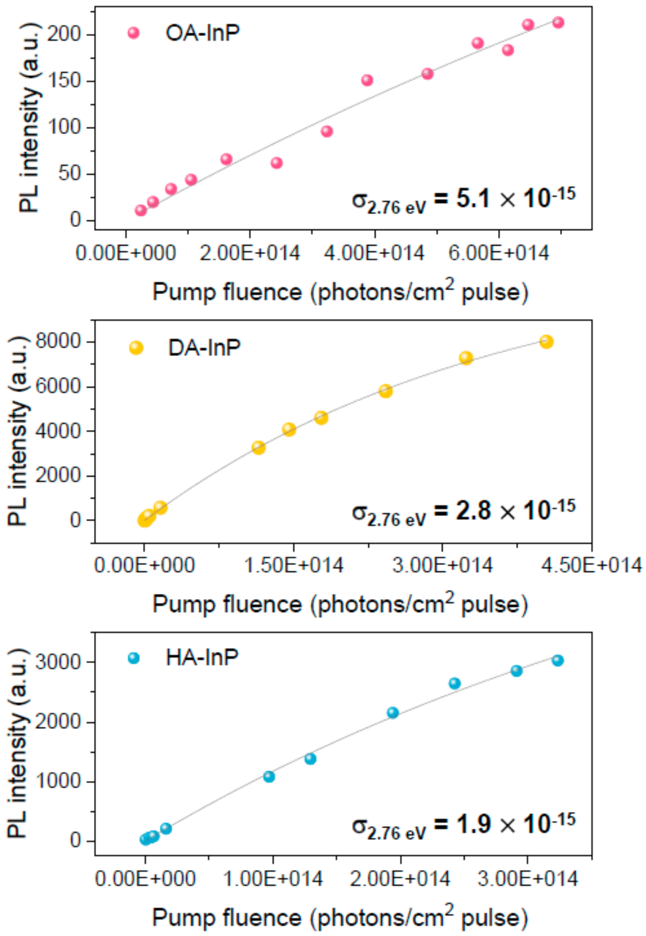

Figure S2. The photoluminescence intensity monitored at t = 1.5 ns, which is when Auger recombination is completed. Data were fitted with the Poisson statistics. After the lifetime of initial decay of multiexciton, the black solid line indicates fits to $(1-p_0)$ calculated for the Poisson distribution of initial QD occupancies [49]. These fitted lines were used to determine the absorption cross-section (σ). Obtained absorption cross sections at 2.76 eV are provided as an inset of the Figure (unit: cm^2).

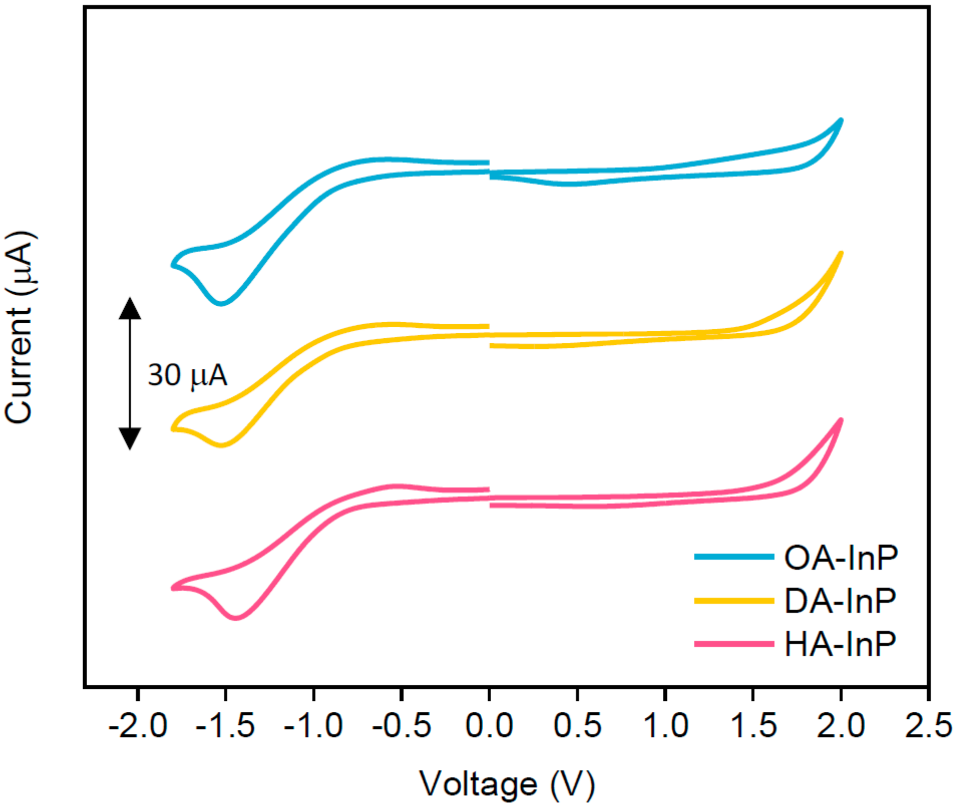

Figure S3. Cyclic voltammograms recorded on OA-InP, DA-InP and HA-InP QDs/dichloromethane containing 0.05 M $TBAPF_6$. Scan rates were 200 mVs^{-1}.

Table S1. Electrochemical bandgap energies obtained by cyclic voltammogram and optical bandgap energies obtained by absorption spectra.

	V_{red}	V_{ox}	E_{elec}	E_{opt}
**OA-InP**	−0.86 V	1.61 V	2.47 eV	2.26 eV
**DA-InP**	−0.83 V	1.63 V	2.46 eV	2.27 eV
**HA-InP**	−0.86 V	1.63 V	2.49 eV	2.26 eV

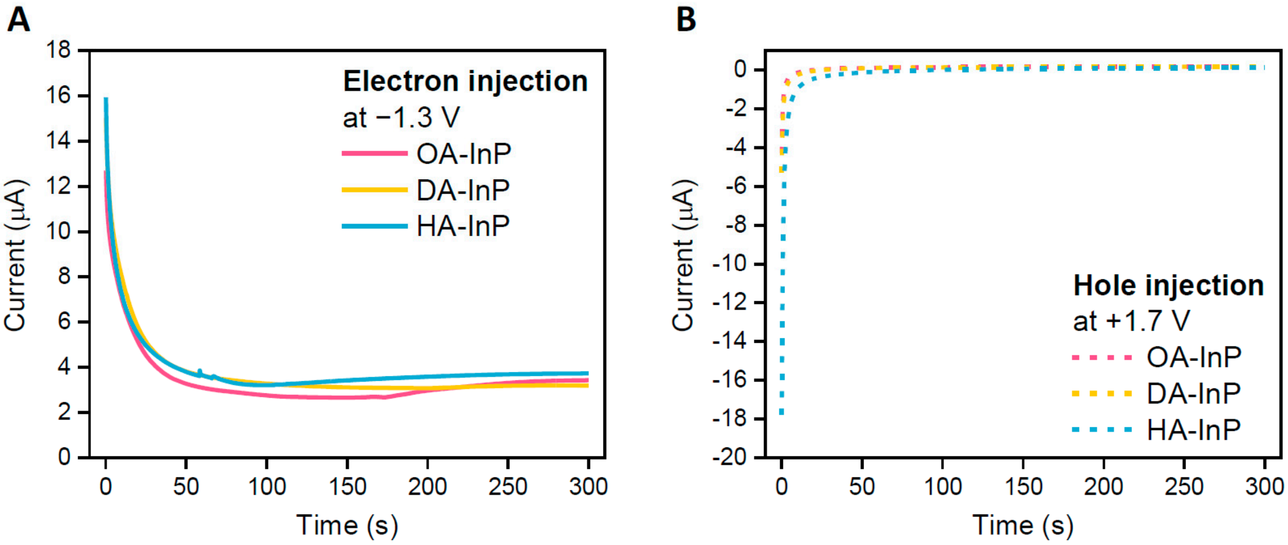

Figure S4. Chronoamperometry measurement at (**A**) −1.3 V and (**B**) +1.7 V.

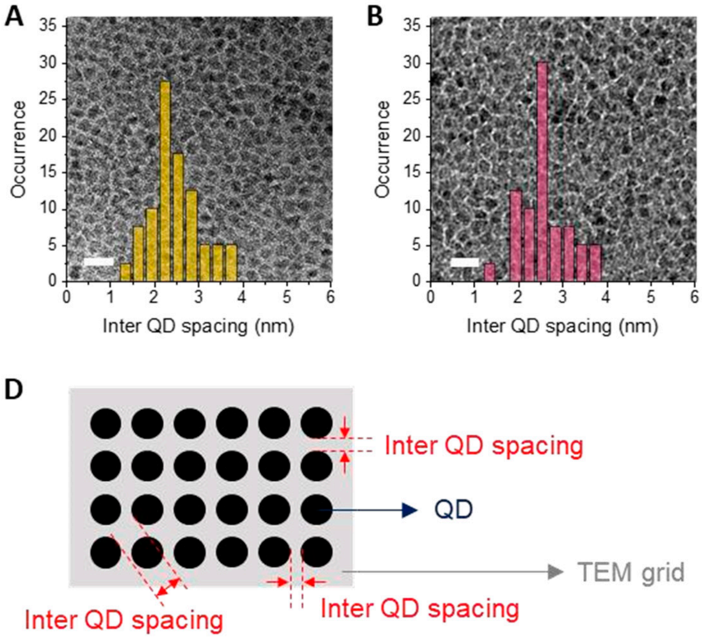

Figure S5. Transmission electron microscopy (TEM) images and corresponding histogram of inter QD spacing of (**A**) OA-InP, (**B**) DA-InP and (**C**) HA-InP packing on a TEM grid (Scale bars: 20 nm). (**D**) Measurements of the inter QD spacing from TEM images using Image J Software (Image J, version 1.x, National Institutes of Health and the Laboratory for Optical and Computational Instrumentation, Bethesda, MD, USA).

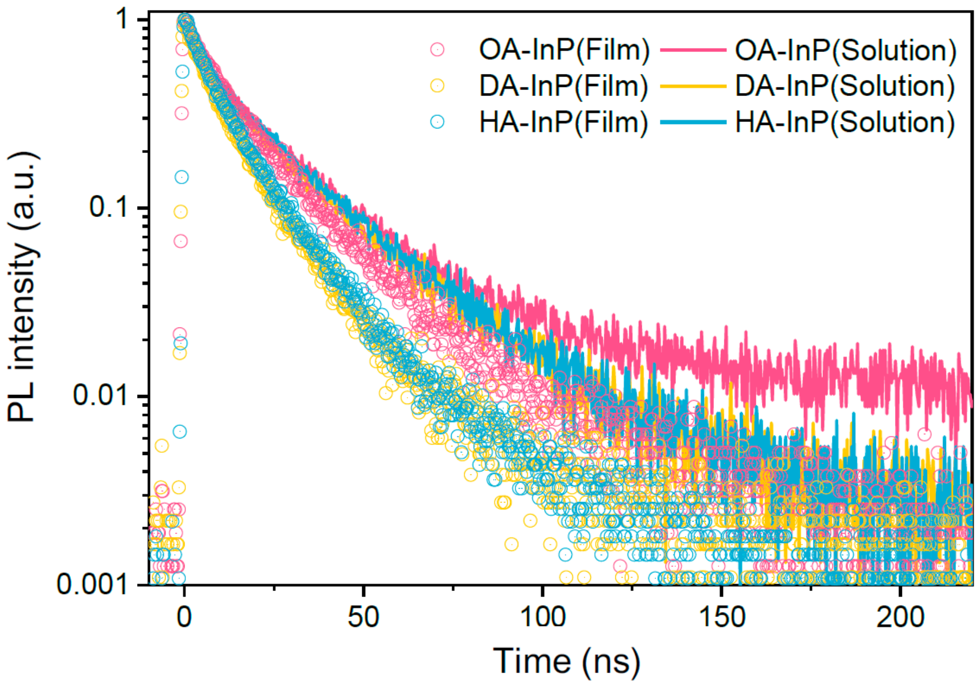

Figure S6. The photoluminescence decay curves of OA-InP, DA-InP and HA-InP solution and solids.

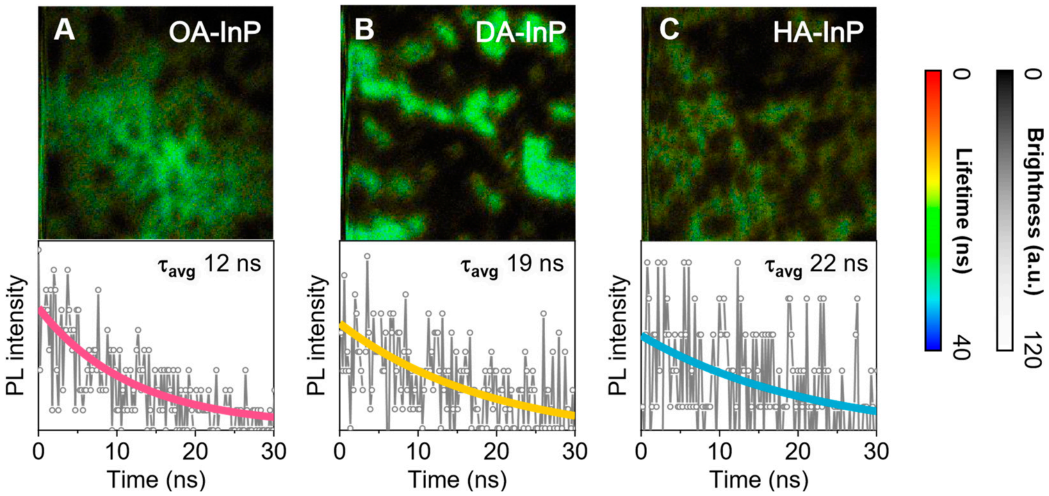

Figure S7. (**A–C**, top) Photoluminescence lifetime-intensity images of disordered InP/ZnSe/ZnS films (Scale bar: 1 μm). (**A–C**, bottom) Corresponding averaged photoluminescence lifetimes of disordered InP/ZnSe/ZnS films.

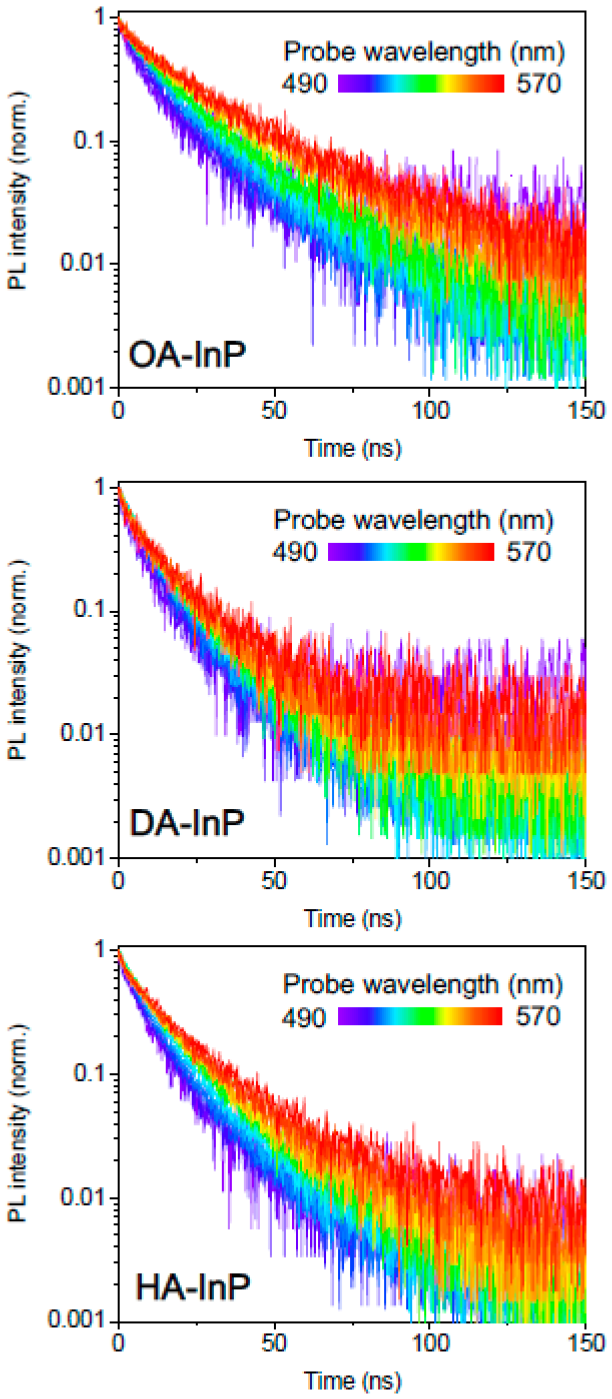

Figure S8. The photoluminescence decay curves of InP/ZnSe/ZnS QD solids at the wavelengths corresponding to the colored bar in the inset.

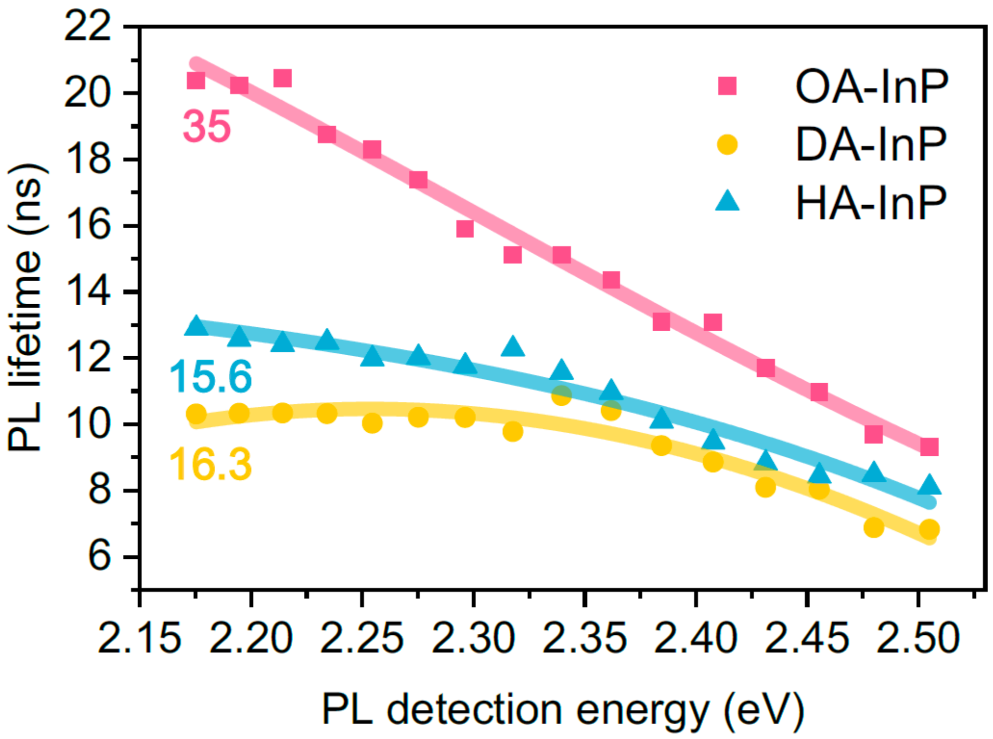

Figure S9. The photoluminescence lifetimes as a function of detection energy (Inset: fitting parameters representing the rate of increase of the PL lifetime with the detection energy).

Table S2. FRET efficiency for OA-InP, DA-InP and HA-InP.

Parameter	OA-InP	DA-InP	HA-InP
Orientation factor, κ	0.816	0.816	0.816
Photoluminescence quantum yield, ϕ	0.88	0.76	0.80
Spectral overlap integral, J(λ) [nm4/M*cm]	2.74 E+16	1.42 E+16	1.37 E+16
Refractive index, n	2.41	2.41	2.41
Critical distance, R_0 [nm]	5.9	5.1	5.1
Effective shell radius, R_S [nm]	~1.9	~1.9	~1.9
Spacing between QDs, R_L [nm]	2.6	2.5	2.4
Distance between QDs, r [nm]	6.4	6.3	6.2
FRET efficiency, E_f [%]	38	23	23

Supporting Notes for Calculation of the FRET Efficiency

R_0 is calculated using $R_0 = 0.211\left(\frac{\kappa^2\phi * J(\lambda)}{n^4}\right)^{\frac{1}{6}}$; where n is a volume-weighted average of the refractive indices of InP, ZnSe, ZnS, and an organic ligand [35,50]. E_f is calculated as $E_f = \frac{R_0^6}{R_0^6 + r^6}$; where $r = 2R_S + R_L$. R_L is directly measured from the high-resolution TEM image of the QD films.

The spectral overlap integral (J), in units of $M^{-1}cm^{-1}nm^4$, is defined by

$$J(\lambda) = \int_0^\infty \varepsilon_A(\lambda)\lambda^4 F_D(\lambda)d\lambda$$

where ε_A is the extinction coefficient spectrum of the acceptor, F_D is the wavelength-dependent donor emission spectrum normalized to an area of 1.

The spectral overlap integral under the absorption and emission spectra was calculated using the UV-Vis-IR Spectral Software, as shown in Figure below (a|e, version 2.2, http://www.fluortools.com/home (accessed online: 20 February 2023), Copenhagen, Denmark, 2015).

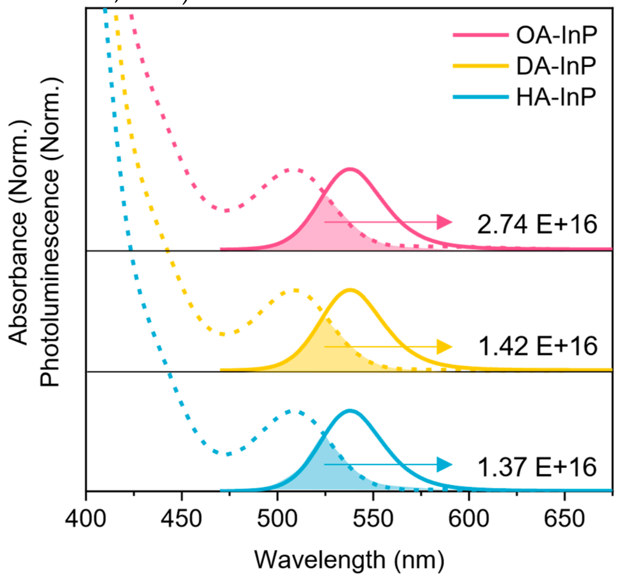

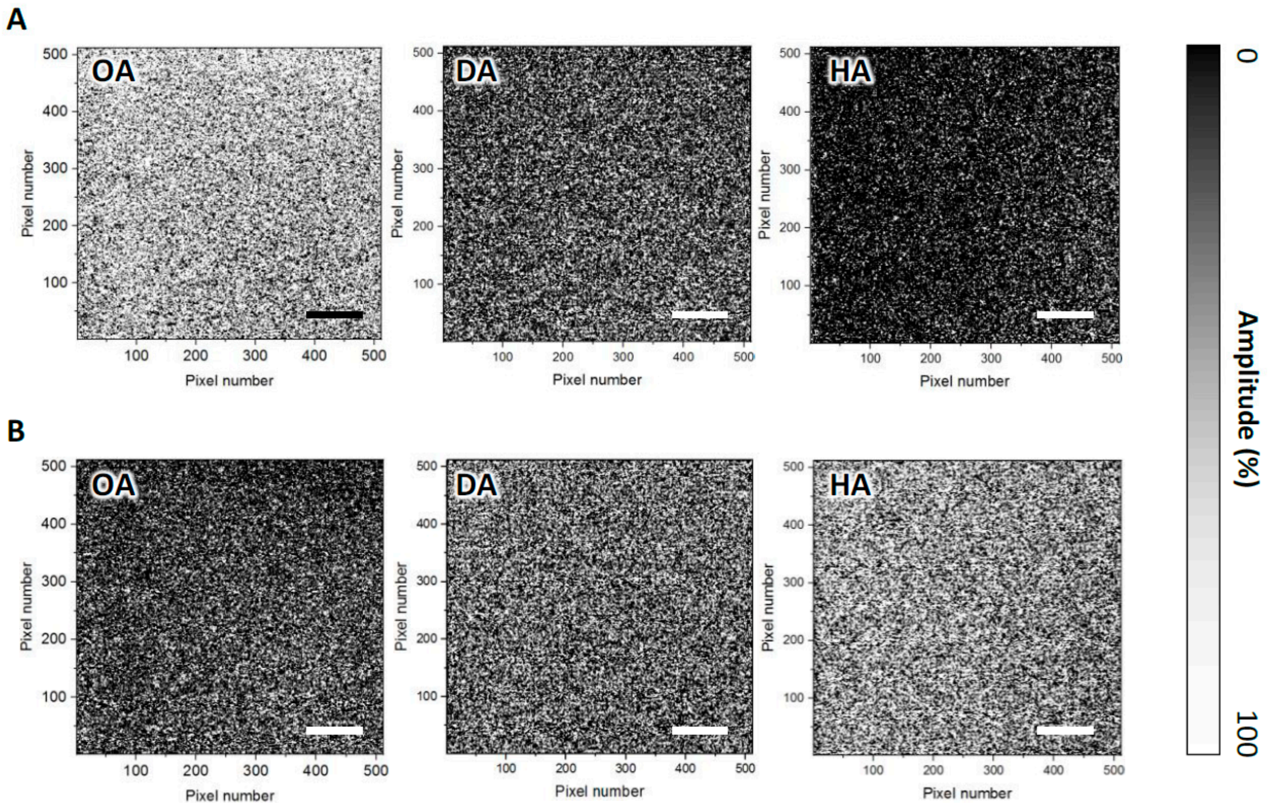

Figure S10. (A) The images of A_1 and (B) A_2 in OA-InP, DA-InP and HA-InP (Scale bar: 1 μm), where photoluminescence decay profiles in FLIM images of OA-InP, DA-InP and HA-InP ordered arrays are fitted using the relation $I(t) = A_1\exp(-t/\tau_1) + A_2\exp(-t/\tau_2)$. I(t) is the time-dependent photoluminescence intensity, A_1 and A_2 the amplitudes, and τ_1 and τ_2 the fitted photoluminescence lifetimes.

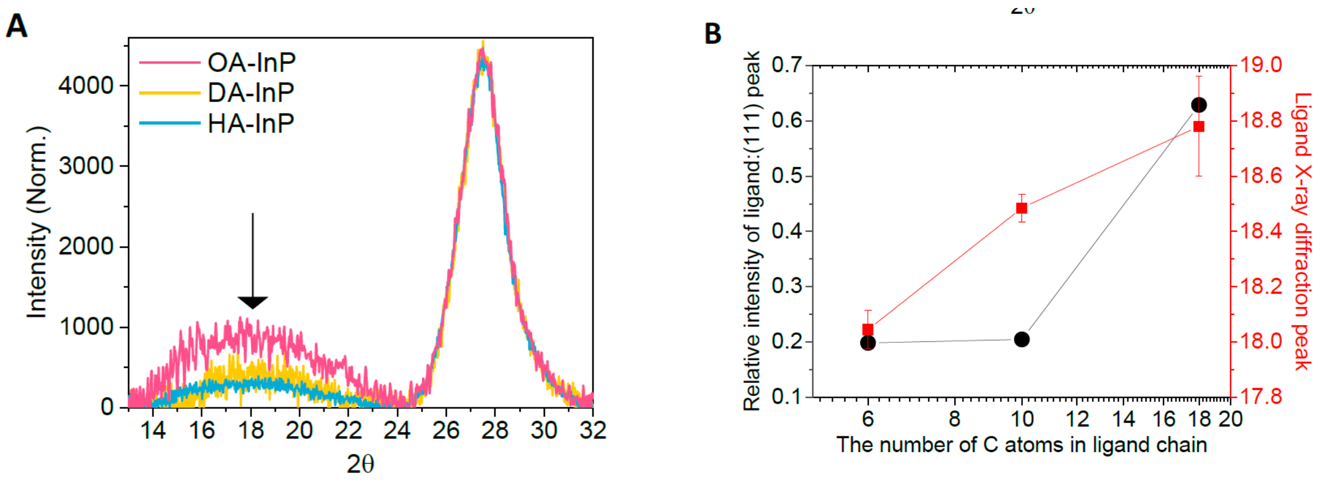

(**a**) (**b**)

Figure S11. (**A**) Aliphatic ligand peak of XRD spectra of OA-InP, DA-InP, and HA-InP. (**B**) Relative intensity of ligand peak to the (111) peak and ligand diffraction peak position of InP/ZnSe/ZnS QDs.

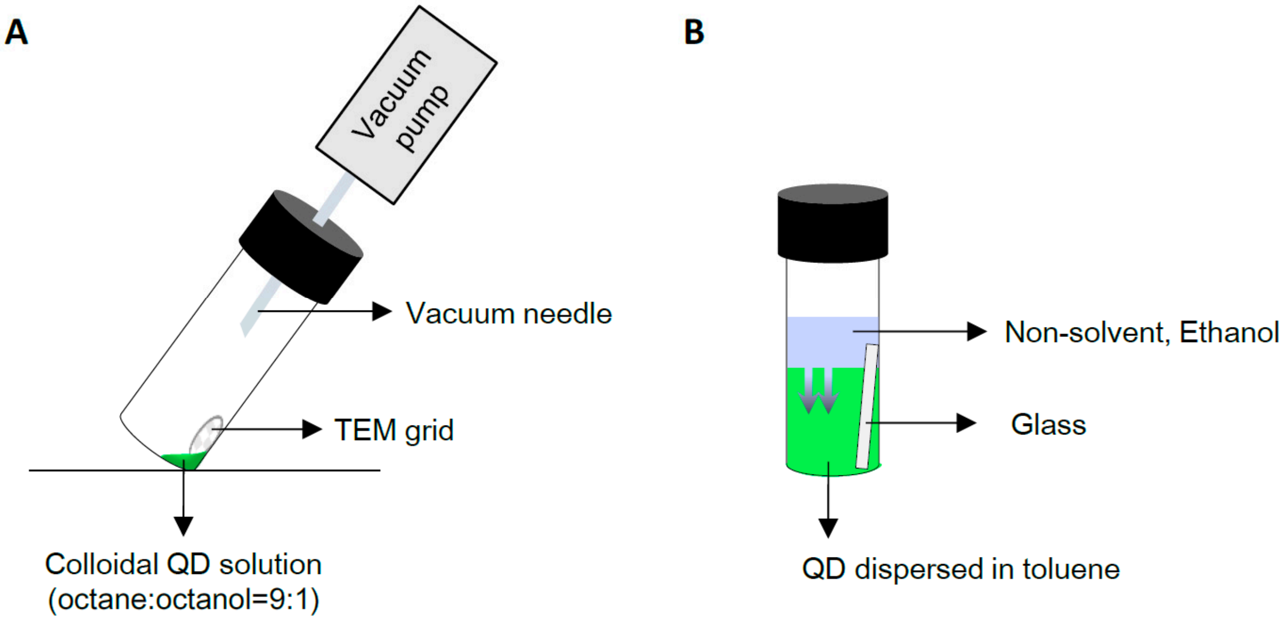

Figure S12. (**A**) Deposition of InP/ZnSe/ZnS QDs on the TEM grid by vaccum assisted evaporation of colloidal QD solution (QD dispersed in octane:octanol = 9:1 volume ratio). (**B**) Growing InP/ZnSe/ZnS QD array on glass substrate by gentle destabilization of the colloidal solution.

References

27. Yoon, S.J.; Guo, Z.; Dos Santos Claro, P.C.; Shevchenko, E.V.; Huang, L. Direct Imaging of Long-Range Exciton Transport in Quantum Dot Superlattices by Ultrafast Microscopy. *ACS Nano* **2016**, *10*, 7208–7215.
35. Kagan, C.R.; Murray, C.B.; Bawendi, M.G. Long-Range Resonance Transfer of Electronic Excitations in Close-Packed CdSe Quantum-Dot Solids. *Phys. Rev. B* **1996**, *54*, 8633–8643.
49. Makarov, N. S.; Guo, S.; Isaienko, O.; Liu, W.; Robel, I.; Klimov, V. I. Spectral and Dynamical Properties of Single Excitons, Biexcitons, and Trions in Cesium-Lead-Halide Perovskite Quantum Dots. *Nano Lett.* **2016**, *16*, 2349–2362.
50. Lakovicz, J. R. *Principles of fluorescence spectroscopy*; Springer, Germany, 2006.
